# Conditioned medium derived from bovine umbilical mesenchymal stem cells as an alternative source of cell-free therapy

**DOI:** 10.14202/vetworld.2021.2588-2595

**Published:** 2021-10-05

**Authors:** Dwi Liliek Kusindarta, Hevi Wihadmadyatami

**Affiliations:** Department of Anatomy, Faculty of Veterinary Medicine, Universitas Gadjah Mada, Yogyakarta, 55281, Indonesia.

**Keywords:** bovine umbilical cord blood, cell-free therapy, mesenchymal stem cells, secretome

## Abstract

Umbilical cord blood (UCB) cells are an important source of mesenchymal stem cells (MSCs). It is known that the umbilical cord is rich in hematopoietic stem cells, which influenced research on ontogeny and transplantation (allogeneic transplantation). In recent years, stem cell research has emerged as an area of major interest due to its prospective applications in various aspects of both human and veterinary medicine. Moreover, it is known that the application of MSCs has several weaknesses. The use of these cells has limitations in terms of tumorigenesis effect, delivery, safety, and variability of therapeutic response, which led to the use of secretomes as an alternative to cell-free therapy. The main obstacle in its use is the availability of human UCB as an origin of MSCs and MSCs’ secretomes, which are often difficult to obtain. Ethical issues regarding the use of stem cells based on human origin are another challenge, so an alternative is needed. Several studies have demonstrated that MSCs obtained from bovine umbilical cords have the same properties and express the same surface markers as MSCs obtained from human umbilical cords. Therefore, secretomes from MSCs derived from domestic animals (bovine) can possibly be used in human and veterinary medicine. This finding would contribute significantly to improve cell-free therapy. At present, the use of UCB MSCs derived from domestic animals, especially bovines, is very restricted, and only limited data about bovine UCB are available. Therefore, the aim of this review was to provide an updated overview of cell-free therapy and discuss the new possibilities introduced by the generation of this therapy derived from bovine umbilical MSCs as a promising tool in developing modern and efficient treatment strategies.

## Introduction

In recent 20 years, stem cell research has emerged as an area of significant interest for its potential applications in both human and veterinary medicine. Mesenchymal stem cells (MSCs) are omnipresent in various tissues and are not limited to mesodermal origins such as bone marrow, adipose, muscle, or bone. These cells can also be found in the brain, spleen, liver, kidney, lung, thymus, umbilical, and pancreas. Recently, MSCs derived from umbilical cords have been broadly accepted in the field of both human and veterinary medicine for the treatment of degenerative diseases (e.g., osteoporosis, osteoarthritis, diabetes mellitus, dementia, Alzheimer’s disease, and Parkinson’s disease) and for beauty-related therapies. In veterinary medicine, stem cells are also widely used for the treatment of wounds, reproductive problems, cardiac diseases, diabetes, infertility, etc. The use of umbilical cord blood (UCB) transplantation has shown a decrease in rejection, as UCB cells have low human leukocyte antigen levels [1]. Notably, implanted UCB cells have not evidently shown to form teratomas, unlike embryonic stem cells [2].

However, there are several critical problems related to safety that are necessary to solve when choosing MSCs for the treatment of humans. The potential of MSC-based therapy for tumorigenicity has been recently reported. Despite the many clinical results and ongoing clinical trials, there are no clear reports on the long-term safety of MSCs, including human UCB (HUCB)-MSCs. In addition, the ­presumed profibrogenic potential of MSCs, as demonstrated in human bone marrow-induced liver fibrosis, poses another obstacle to their therapeutic use. Moreover, MSCs are also a large heterogeneous mixture of various cell populations defined by cell surface phenotypes; their functions can be differentiated into multiple cell descendants, such as osteoclasts, chondrocytes, adipocytes, and myocytes. Heterogeneity was found within and among MSC populations from various sources, including donor, tissue, clonal, and single-cell subpopulations, according to age, sex, genetics, environmental conditions, aging, and epigenetic modification. Recently, the angiogenetic proportion of HUCB-MSCs used for treatment is highly donor-dependent [3,4]. The delivery, safety, and variability of the treatment response are several hindrances for the use of HUCB-MSCs, which led to the use of secretomes as a substitute to cell-free therapy at the beginning of the 20^th^ century [5].

The other main obstacle is the availability of human umbilical cords, which are often difficult to obtain and are highly associated with bacterial and fungal contamination. There are also ethical restrictions on the use of stem cells of human origin. A substitute for the human umbilical cord as a source of MSCs and secretomes is needed. Some studies have shown that MSCs obtained from bovine umbilical cords have the same properties and express the same markers as these cells obtained from human umbilical cords. Thus, secretome products from bovine umbilical MSCs (BUMSCs) can possibly be used in human and veterinary medicine. This finding would contribute significantly to improve cell-free therapy. However, since the use of umbilical cords of domestic animals, especially bovines, is very restricted, only limited data about it are available (recent data only provide stem cells derived from rat umbilical cords). Therefore, this review aimed to contribute to an updated overview of cell-free therapy using BUMSCs and discuss new future possibilities of cheap, hopeful, and efficient treatment strategies. Moreover, this review discussed the structure of the bovine umbilical cord and the components and advantages of BUMSCs, including the conditioned medium (CM) derived from BUMSCs, as a new therapy for neurodegenerative diseases.

## The Structure of Bovine Umbilical Cords

**Figure-1:**
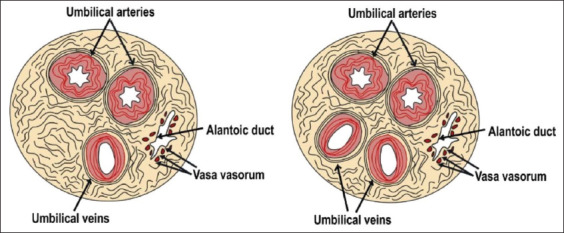
Cross-section of the human umbilical (left panel) and bovine umbilical cord (right panel). The schematic diagram shows the main similarities between these umbilical cords, consisting of an artery and a vein enclosed in Wharton’s jelly.

The umbilical cord is a tube-like structure that connects the placenta and the fetus, and it plays a significant role in the interaction between the mother and fetus during pregnancy. This structure maintains the viability and facilitates the growth of the embryo and fetus. The umbilical cord is very important for the fetus’ development, well-being, and survival. It provides oxygen and nutrients and eliminates waste products. The UCB vessels have different structures and functions compared to other blood vessels in the body. Umbilical arteries drain blood from the fetus to the placenta, while umbilical veins work the other way around. The umbilical cord’s length increases in line with the gestational age, and its average length depend on the length of the fetus itself. The umbilical cord’s outer layer consists of the amniotic ­epithelium, while its inner layer has an internal mesodermal mass called Wharton’s jelly. Wharton’s jelly comes from the mesenchymal layer, which is composed of collagen, hyaluronic acid, muscle fibers, and water. This structure provides mechanical support and structural protection for the umbilical cord. It also has angiogenic and metabolic roles in umbilical cord circulation. Wharton’s jelly has two endodermal ducts (allantois duct and vitelline duct) and umbilical blood vessels. In humans, a normal umbilical cord consists of two umbilical arteries and one umbilical vein and is surrounded by Wharton’s jelly and a single layer of amniotic membrane. In domestic animals, such as cattle, sheep, and buffalo, the umbilical cord consists of two umbilical arteries and two umbilical veins (Figure-1).

Under an electron microscope, the structure of bovine UCB (BUCB) cells is morphologically compatible with that of the precursor of blast cells. Erythrocytic, neutrophilic, eosinophilic, basophilic, monocytic, and lymphocytic lineages were observed, along with three different cytoplasmic granules of bovine neutrophils. Uncommon cells with peculiar morphological features resembling apoptotic cells were also found. Mature cells were also observed with the morphological features of apoptotic processes. The existence of a huge number of immature cells in HUCB has also been reported [6]. In this study, granulocytes, including band cells, basophils, eosinophils, and neutrophils, showed ultrastructural characteristics similar to those in humans, as described by some authors [7-10]. Human and UCB cells have similar structures, suggesting that bovine umbilical cords could constitute a model for human umbilical cord research and medication-based therapy for stem cell and cell-free therapy.

## The Human and BUMSCs and the CM

**Figure-2:**
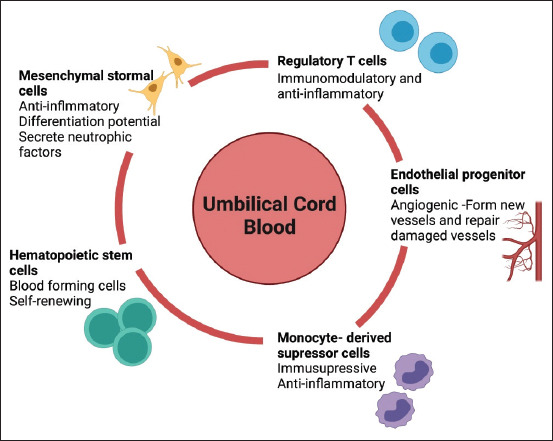
The main stem and progenitor cells present in umbilical cord blood (UCB) and their primary functions. The diagram represents the five main stem and progenitor cell subtypes produced by UCB, which include mesenchymal stromal cells, endothelial progenitor cells, hematopoietic stem cells, monocyte-derived suppressor cells, and regulatory T cells.

Today, MSCs are considered the most popular choice of treatment and part of tissue engineering material. Multiple studies have shown that the roles of MSCs in regeneration are mostly facilitated by their capacity to produce a wide range of bioactive chemicals (Figure-2).

Several data showed similar surface markers found in MSCs derived from human and BUCB (Table-1) [11-16].

**Table-1:** Surface markers of human umbilical mesenchymal stem cell and bovine umbilical mesenchymal stem cell.

Marker	Human Umbilical Mesenchymal Stem Cell	Bovine Umbilical Mesenchymal Stem Cell
CD 70	+ [11]	−
CD 90	+ [12]	+ [15]
CD 105	+ [13]	+ [15]
CD 29	+ [14]	+ [15]
CD 44	+ [11]	+ [16]
CD 73	+ [14]	+ [16]
CD 90	+ [14]	+ [16]
CD 166	+ [11]	+ [16]

+=Present; -=Not found

Secretome was first described in the early 20^th^ century. Gnecchi *et al*. [17] introduced the therapeutical effects of MSCs mediated by the secretion and release of trophic molecules. Recently, these trophic molecules have become known as secretome [5]. Since secretome, microvesicles, and exosomes are found in a culture medium where MSCs are cultivated, the latter is called CM [18]. The use of MSC-CM has several benefits compared to the use of stem cells. The CM, which is a complex factor produced in cell culture growth medium as a well-defined biopharmaceutical drug, can potentially be a direct MSC treatment substitute [19].

### What is inside the BUMSCs CM?

**Figure-3:**
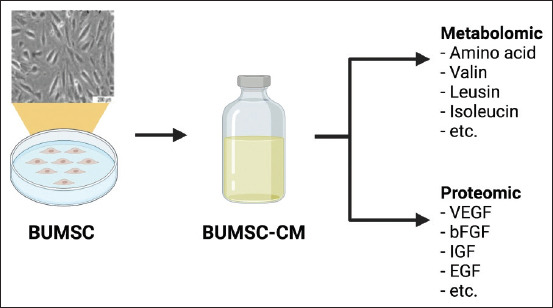
Bovine umbilical mesenchymal stem cells (BUMSCs) and its conditioned medium (BUMSC-CM). The first passage is usually the best conditioned medium, which is the ingredient with growth factors, cytokines, amino acids, and other elements that can be identified using metabolomic and proteomic techniques.

The MSCs’ secretome is a complex mixture of dissolved products consisting of soluble protein fractions (growth factors and cytokines) and vesicular fractions (microvesicles and exosomes). Both soluble protein and vesicular fractions involve the transfer of proteins and genetic substances (e.g., microRNA) to other cells, with a prospective therapeutic effect [20]. The MSCs’ secretome is considered a potent active pharmaceutical component, and its vesicular properties are promising for use on a drug delivery system. Primarily because of its traceability, it opens windows of opportunities to reach specific targets and compound targets (e.g., drugs and proteins) that are released directly into affected organs (lesions) [21]. In detail, secretome has shown numerous chemokines and growth factors (e.g., epidermal growth factor [EGF], Vascular Endhotelial Growth Factor [VEGF], fibroblast growth factor [FGF], platelet-derived growth factor [PDGF], hepatocyte growth factor [HGF], transforming growth factor [TGF], tumor necrosis factor [TNF], stromal cell-derived factor 1 [SDF-1a], interleukin [IL]-6, IL-8, and insulin-like growth factor [IGF]). It also includes adhesion molecules, receptors, and metalloproteinase substances, which involve the MSCs’ migration process (e.g., MMPs, CXCL-12, CCL-2, CCL-3, CCR4, CXCR4, VCAM, ICAM, PECAM, and ADAM-12; Figure-3).

Some studies have reported that VEGF, basic FGF (bFGF), IGF-1, and EGF are growth factors that participate in endothelial cell development and hematopoietic differentiation. The CM derived from UCB cells supported the production of mesodermal precursor cells and several kinds of hematopoietic progenitor cells. The effect was as vigorous as the effect of a growth factor mixture (a combination of VEGF, FGF, IGF, and EGF), which proposes that the growth factors in MSCs’ secretome could also promote the differentiation of embryonic stem cells into endothelial and hematopoietic cells [22]. The findings of this study using the metabolomic approach (Liquid Chromatography-Mass Spectrometry/Mass Spectrometry) revealed that BUMSCs contain many amino acids (Table-2). The amino acids in the secretome also contain some glucose (a-glucose and b-glucose), vitamins, nicotinamide, and end-products of choline and amino acid oxidation [23].

**Table-2:** The main metabolite present in the bovine umbilical mesenchymal stem cell conditioned medium.

Metabolite	Abbreviation
Isoleucine	Ile
Leucine	Leu
Valin	Val
Ethanol	Et
Threonine	Thr
Lactate	Lac
Alanine	Ala
Lysine	Lys
Arginine	Arg
Acetate	Ace
Glutamate	Glu
Glutamine	Gln
Methionine	Meth
Choline	Cho
Pyruvate	CH_3_
a-Glucosa	a-Glu
b-Glucosa	b-Glu
Tyrosine	Tyr
Phenylalanine	PheAla
Histidne	His
Tryptophan	Try

In addition to the analyzed the secretome’s growth factor, study with NCBI blast (https://blast.ncbi.nlm.nih.gov/Blast.cgi) was done, this study aim to understand the homology between several growth factors present in HUMSCs and BUMSCs conditioned media. There was 80-99% homology between them (Table-3).

**Table-3:** Percent identity (homology) of the growth factor that is present on the secretome derived from a human umbilical and bovine umbilical mesenchymal stem cell (the alignment and blast are performed using NCBI Blast for Protein).

Name of Protein	Accession Number Human	Accession Number Bovine	Homology
VEGF	AAP86646.1	AAA30502.1	83.90%
FGF	2AXM_A	NP_001300935.1	92.59%
TGF	AAI04564.1	AAG43048.1	99.62%
TNF	EAX03424.1	AAI34756.1	80%

TGF=Transforming growth factor, VEGF=Vascular endhotelial growth factor, FGF=Fibroblast growth factor, TNF=Tumor necrosis factor

### Advantages of MSCs CM (secretome)

Despite the lack of MSC in its end formula, the remarkable therapeutic potency of stem cell CM makes it recognized as “cell-free therapeutics.” At present, cell-free therapy (secretome) provides a significant advantage over stem cell transplantation. There are many benefits that secretome can provide as free therapy cells. First, using cell-free products based on biologically active factors produced by stem and progenitor cells can notably decrease the risks that are results of injecting cells directly. Second, secretome compounds produce lower cell surface proteins, providing lower immunogenicity in contrast to living and proliferative cells [24]. Third, secretome, as a ready-made product, considerably reduces the number of cells required for transplantation (7×10^6^ cells/kg). Fourth, phenotypic changes and therapeutic potentials are not possible due to prolonged MSC expansion *in vitro* before transplantation. Fifth, higher production rates are possible using dynamically controlled laboratory conditions (e.g., bioreactors) [20]. Sixth, a CM structure for secretome can be chosen for clinical usage due to its procedure, which circumvents the invasive cell collection; hence, it becomes considerably more economical and practical. Seventh, MSC secretions acquired for therapeutic applications can be altered to produce a cell-specific effect, and it is not compulsory to have matching donor and recipient in order to prevent rejection issue. Eighth, the time and cost of developing and maintaining cultivated stem cells can be significantly reduced, and ready-to-use secretory therapies are readily available for treatment. Ninth, the assessment of safety, dose, and potency of MSC secretions can be done in a similar way using standard pharmaceutical compounds. Finally, secretome storage can be carried out safely (e.g., freeze-dried) and without the loss of product potency by eliminating the use of potentially toxic cryoprotective agents [24,25].

Many studies have shown that a CM can serve as a treatment for various diseases, such as acute and chronic wound healing, myocardial infarctions, cerebral injuries, strokes, degenerative diseases, bone defects, and lung injuries. Data showed that medication using a CM has promising; for example, patients with chronic kidney diseases treated using a human embryonic stem cell-derived MSCs CM had decreased systolic blood pressure and proteinuria [25].

Harvesting a CM can be done from multiple cell types. Various methods can be chosen to obtain a CM, which may interfere with the methods to optimize the level and type of growth factors, chemokines, cytokines, adhesion molecules, and other aspects. Only a few studies examined the growth factor levels when using a CM. The diversity of cell numbers, culture media, and conditions, including the CM processing, affect different growth factor levels in the same cells. There are few factors involved in the CM or secretome quality. The first is the culture medium–the use of fetal bovine serum or another supplement containing the complete medium. In contrast, different studies may not use serum-free media. An *in vitro* culture basically represents *in vivo* microenvironment conditions that may influence the fate of cells and their secretions. Consequently, the use of different media for the same cell might result in different growth factors. The second factor is the culture duration. Various durations can be referred to when producing a CM. Some research describes the range of culture duration from 16 h to 5 days. In using a complete medium, a shorter culture period might leave some serum-acquired growth factors that were not consumed by cells, increasing the levels of growth factors or suppressing the secretion of growth factors by cells. The last factor is the culture condition. Several studies have produced a CM from cell cultures in normoxic (O_2_ level: 20-21%) and hypoxic (O_2_ level: 0.5%, 1%, 1.5%, and 2%) conditions.

The previous studies on various types of stem cells have shown the upregulation of major growth factors in hypoxic conditions such as VEGF, HGF, PDGF, and except EGF, which was downregulated. Recently, almost all studies have proposed the use of monolayer cultures instead of spheroid cultures to produce a CM. The main reason for this choice is that spheroid cultures require particular handling and apparatus (e.g., spinner flask). Moreover, an increase in the levels of secreted factors was observed when choosing spheroid cultures due to their ability to yield more cells than standard monolayer cultures. Moreover, cells can be in a hypoxic state within the center of a spheroid culture, unlike cells on the surface. A hypoxic condition will increase the growth factor yield.

## The Role of BUMSC CM on Neurodegeneration

Conditioned media consist of growth factors, pro- or anti-inflammatory cytokines, and other molecules, including leptin, angiogenin, granulocyte-macrophage colony-stimulating factor, monocyte chemoattractant protein-1, SDF-1 or CXCL12, and fractalkine [25,26]. VEGF plays a significant role in the formation of immature vessels (vasculogenesis) [27] and angiogenesis, and it enhances vascular permeability [28]. A rat model with Parkinson’s disease has demonstrated the role of VEGF in bringing about neuro-proliferation or neuro-rescue events [29]. The survival and functionality of striatal neurons are due to brain-derived neurotrophic factors (BDNFs), which are stem cell-secreted cytokines [30]. An overexpression of BDNFs indicates the survival and increased synaptic markers of neurons of an Apolipoprotein P (APP/PS1) transgenic mouse model with Alzheimer’s disease [31,32]. BDNFs and glial-derived neurotrophic factors (GDNF) increase the functional outcomes of and recovery from strokes [33]. SDF-1 acts as the attachment of cells into an ischemic area [34]. Nestin and neural growth factors (NGFs) are known to uplift the neurogenic effects and neuroprotective actions that are exhibited in a host [35]. Research has demonstrated the effectiveness of IL-6 for neuroprotection as a result of pro- or anti-inflammatory factors [36]. It was also proposed that a CM has a great anti-inflammatory, anti-apoptotic, and antioxidant potential, and it may moderate the reconstruction of cerebral architecture (e.g., through vascular remodeling or re-establishment of neuronal networks). In various unfavorable conditions, these benefits could play a major role in cerebral repair.

**Figure-4:**
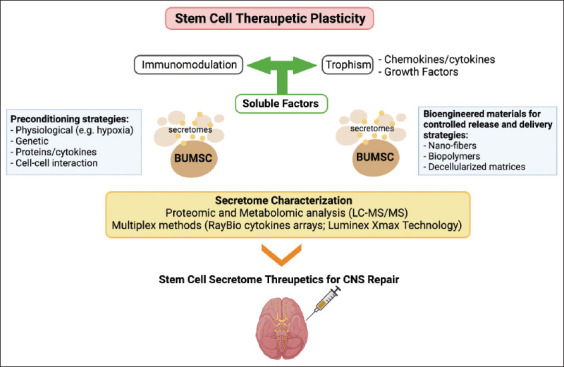
Proposed model for the therapeutic application of mesenchymal stem cell secretome for central nervous system (CNS) repair. The exchange of signals between grafted bovine umbilical mesenchymal stem cell and the host leads to remarkable tissue trophic effects on endogenous brain cells and beneficial modulatory actions on innate and adaptive immune responses, which ultimately promote the healing of the injured CNS [41].

The previous studies have shown that VEGF, BDNF, GDNF, SDF◻1, IGF◻1, and HGF are responsible for therapeutic efficacy of cultured media for strokes. In the recovery of patients with Parkinson’s disease, BDNF, CNTF, FGF◻2 (bFGF), FGF◻8, FGF◻20, GDNF, IGF◻1, IL◻6, NGF, SDF◻1a, TGF◻b1, TNF◻a, TRKC, and VEGF are present [37]. The secretions of growth factors are responsible for the neurotrophic, neuroprotective, and immunomodulatory effects in Parkinson’s disease [38-40], stroke [33], Huntington’s disease [41], and Alzheimer’s disease [32]. Growth factors and cytokines may reduce oxidative stress, increase the amount of angiogenesis for neurogenesis, and increase the migration, differentiation, and survival of endogenous cells (Figure 4) [42].

## Future Prospectus

In the last few decades, cell-free therapy has become one of the popular therapies that aimed to overcome the therapeutic problems arising from the administration of stem cells, especially those related to autologous cell production and immunological rejection [43]. At present, the use of stem cells or secretomes from humans faces problems, especially in terms of ethics, law, and policy issues. Moreover, it becomes interesting for scientists to develop other alternatives as sources of stem cells and secretomes, which in this review are bovine. The use of bovine umbilical cord as a source of stem cells and secretomes still requires much integrated research in multi-disciplinary fields of science, both at the level of basic science (fundamental) to its application in the clinical or translational phase for medicinal purposes, especially for degenerative diseases. At present, the use of a CM gives hope for many untreatable degenerative diseases, such as neurodegenerative diseases (Parkinson’s disease, Huntington’s disease, and motor neuron disease), macular degeneration, which can possibly become treatable with stem cell and cell-free therapy [44-46]. This condition encourages researchers to develop many sources to acquire stem cells and CM. In addition, the standardization of the isolation, production process (good manufacturing production), and quality of CM and stem cells are very important, considering the variations in the expression of substances secreted by CM from each production batch, including pro-inflammatory cytokines, anti-inflammatory cytokines, and growth factors. The determination of the dose of CM to use is still a big job that must be completed to optimize its healing ability.

## Conclusion

The use of stem cells and CM for cell-free therapy has been considered as a promising source of treatment, especially for degenerative diseases. BUMSC/CM is known to contain similar markers and products produced by HUMSC/CM, making it possible to utilize BUMSC/CM as an alternative to HUMSC/CM. This review proposed the use of CM or secretome obtained from BUCB as an alternative therapy based on cell-free therapy, especially for patients with regenerative (neurodegenerative) diseases. However, published studies related to the production, characterization, and preclinical and clinical tests of BUMS/CM are still minimal, and when compared to studies on stem cells derived from laboratory animals, such as rats and mice, the data are very limited. Therefore, more profound studies on the use of BUMSCM (secretome) are still needed.

## Authors’ Contributions

DLK: Conception of the review, HW: Literature study and wrote the concept of the original manuscript. HW and DLK: Drafted, reviewed, and edited the final manuscript. Both authors read and approved the final manuscript.
